# Development and psychometric evaluation of a decision quality instrument for lung cancer screening decisions (DQI-LCS)

**DOI:** 10.1186/s12911-026-03346-y

**Published:** 2026-02-21

**Authors:** Naomi Q. P. Tan, Maria A. Lopez-Olivo, Tito R. Mendoza, Laura C. Crocker, Karen R. Sepucha, Shawn P. E. Nishi, Robert J. Volk, Lisa M. Lowenstein

**Affiliations:** 1 Rutgers Cancer Institute, New Brunswick, NJ USA; 2 Division of Medical Oncology, Robert Wood Johnson Medical School, Rutgers University, New Brunswick, NJ USA; 3 Department of Health Services Research, University of Texas MD Anderson Cancer Center, Houston, TX USA; 4 Office of Patient-Centered Outcomes Research, Center for Cancer Research, National Cancer Institute, Bethesda, MD USA; 5 Health Decision Sciences Center, Massachusetts General Hospital, Harvard Medical School, Boston, MA USA; 6 Department of Internal Medicine, Division of Pulmonary Critical Care and Sleep Medicine, The University of Texas Medical Branch, Galveston, TX USA

**Keywords:** Lung cancer screening, Decision quality instrument, Psychometric evaluation, Quality measure

## Abstract

**Background:**

Lung cancer screening (LCS) reduces lung cancer mortality among high-risk individuals. As the screening procedure has both potential benefits and harms, the Centers for Medicare & Medicaid Services mandates a shared decision-making (SDM) discussion prior to the initial LCS. We evaluated the psychometric properties of a decision quality instrument for LCS (DQI-LCS) that assesses whether patients (1) were informed about their options (knowledge), (2) made a screening decision in line with their preferences (goal concordance), and (3) underwent an optimal SDM process (measured using the SDM Process measure [SDMP_4]).

**Methods:**

Secondary analysis of survey data with patients who underwent LCS at tertiary care medical centers in the US within 12 months of the survey. Psychometric evaluation examined the DQI knowledge measure for item retention and deletion, feasibility, test-retest reliability, and validity of the three components of the DQI-LCS.

**Results:**

The final sample (*N* = 264) was 48.0% female and had a mean age of 64.8. One knowledge item (KQ13) was dropped as it had a poor index of discrimination, resulting in a 15-item LCS knowledge measure (LCS-15). We also developed a brief knowledge measure with 5 items (LCS-5). Overall, the DQI-LCS was feasible to complete (under 5% missing data for all items). However, only the overall LCS-15 knowledge measure had acceptable test-retest reliability. The SURE score was positively associated with knowledge, demonstrating construct validity of the knowledge component. When there was goal concordance, there was a greater probability of the participant reporting that they would make the same decision again and be screened every year, demonstrating predictive validity of the goal concordance measure. Finally, higher SDMP_4 scores were associated with higher SURE scores, demonstrating predictive validity of SDMP_4. However, we did not find concurrent validity of SDMP_4 with CollaboRATE.

**Conclusions:**

Overall, there is evidence that the DQI-LCS was feasible to complete, had moderate test-retest reliability, and acceptable construct and predictive validity. A limitation is that our sample only included people who had undergone LCS. With further evaluation, the validated DQI-LCS can be a quality metric for assessing whether patients are receiving guideline-concordant care for LCS.

## Background

Lung cancer is the leading cause of cancer death in the US [1]. Lung cancer screening (LCS) can detect lung cancer at an early stage but there are potential harms [2–4]. Hence, the US Preventive Services Task Force (USPSTF) recommends and the Centers for Medicare & Medicaid Services (CMS) requires that patients have a counseling and shared decision-making (SDM) visit prior to screening [5]. A high quality decision about LCS entails the patient (a) being informed about their options and the associated benefits and harms, (b) making a decision that is concordant with the goals and concerns that matter the most to them, and (c) undergoing a high-quality SDM conversation with their clinician or a healthcare professional [6, 7]. This definition of decision quality has been validated by the International Patient Decision Aid Standards (IPDAS) Collaboration [8].

A number of decision quality instruments (DQI) have been developed to assess the quality of health-related decisions [9–12]. However, there is currently no DQI for LCS decisions and there have been calls for more studies evaluating decision quality measures in the context of LCS [10]. To address this gap, the objectives of this study were (a) to evaluate the psychometric properties of a decision quality instrument for LCS (henceforth referred to as DQI-LCS), and (b) to assess the extent to which patients felt informed about their LCS decisions and their decision to undergo LCS was in line with their goals.

## Methods

This paper follows the reporting guidelines from the Consensus-Based Standards for the Selection of Health Measurement Instruments (COSMIN) [13] and the Guidelines for Reporting Reliability and Agreement Studies (GRRAS) [14] (Supplemental Materials). This study was approved by the Institutional Review Boards at The University of Texas MD Anderson Cancer Center (PA14-0072) and The University of Texas Medical Branch (18–0084).

### Development of the DQI-LCS instrument

The three components of the DQI-LCS are: (1) extent to which the patient is informed (knowledge), (2) extent to which the decision was in line with their goals (goal concordance), and (3) the quality of the SDM process. The DQI-LCS instrument is in the Supplemental Materials.

Knowledge of lung cancer and LCS was adapted from a previously validated measure of knowledge of LCS which had 12 items (LCS-12) [15, 16]. LCS-12 has been found to have acceptable reliability and internal consistency, and high test-retest reliability with a 1-month follow-up [16]. In this study, we removed 2 items, adapted some of the original items, and added 6 new items to map to CMS requirements for LCS (total of 16 items). A brief 5-item (LCS-5) measure representing a subset of the full scale was also developed. Table 1 shows the the construct associated with each knowledge item. Goal concordance was measured using a single item asking if patients wanted to be screened. All participants in this study underwent LCS. Quality of the SDM process was assessed using the previously validated 4-item Shared Decision Making Process (SDMP_4) measure [17]. SDMP_4 is a brief patient-reported measure and one of the three performance measures of SDM outcomes endorsed by the National Quality Forum. It has shown acceptable reliability for LCS decisions, [18] and good discriminant and construct validity across various types of health decisions [17].

**Table 1:** Items in the knowledge of lung cancer screening measure (LCS-15)

Item #	Item	Construct
KQ1	What is the best way to lower the chance of developing or dying from lung cancer?	Smoking as a risk factor
KQ2*	How many people with an abnormal LCS result will have lung cancer?	False positive
KQ3*	How often do health professional groups recommend people get screened for lung cancer?	LCS guidelines
KQ4	Where does lung cancer rank as a cause of cancer death in the US?	Lung cancer risk
KQ5	Do health professional groups recommend all current and former smokers be screened for lung cancer?	LCS guidelines
KQ6	Can lung cancer screening suggest that you have lung cancer when you do not?	False positive
KQ7	Can lung cancer screening miss a possible lung cancer?	Test accuracy
KQ8*	Will all tumors found by lung cancer screening grow to be life threatening?	Overdiagnosis
KQ9*	Without screening, is lung cancer often found at a later stage when cure is less likely?	Importance of screening
KQ10	Is screening recommended for someone who quit smoking more than 15 years ago?	LCS guidelines
KQ11	Is screening recommended for someone who has other health problems that may shorten their life?	LCS guidelines
KQ12	Is screening recommended for someone who is not able or willing to be treated for lung cancer?	LCS guidelines
KQ13 *(dropped)*	How much does screening for lung cancer lower the chances of dying from lung cancer?	LCS effectiveness
KQ14*	Can lung cancer screening find other health conditions?	Incidental findings
KQ15	What is the leading cause of lung cancer in the United States?	Smoking as a risk factor
KQ16	How much radiation is a person exposed to through lung cancer screening?	Radiation exposure

*Items included in brief 5-item measure of knowledge of LCS (LCS-5)

Cognitive testing of the DQI-LCS (knowledge, goal concordance, and SDMP_4 items), and the survey instructions were conducted before administering the DQI-LCS with LCS patients [19]. Changes were made iteratively to improve comprehension and ease of completing the survey. Overall, 10 participants participated in 5 rounds of testing and refinement.

### Participants

This was a secondary analysis of data from a cross-sectional survey conducted with patients at two academic tertiary care centers, one an academic medical center and the second a cancer center, in the South Central region of the US [20]. The survey included patients (1) ages 55–80 (in line with USPSTF’s LCS guidelines prior to the 2021 update [21]); (2) who currently or formerly smoked, and (3) had undergone low-dose computed computed tomography (LDCT) for LCS within 12 months of the survey.

### Data collection procedures

Patients at the study sites were invited to participate in the study from May to July 2018. Participating sites were provided with (1) physical study packets which include a cover letter, invitation letter, questionnaire statement, and the survey, and (2) a template of the email invitation letter, the questionnaire statement, and a link to the online survey. Clinic staff at the participating sites identified potentially eligible participants and distributed the study materials. Consecutive patients who met the inclusion criteria for the study were invited to participate in the study. Participants either mailed the completed surveys back to the research team using pre-paid envelopes or completed the survey online on REDCap. A month after the baseline survey (T0) was completed, participants were sent a follow-up survey (T1) which included the same measures.

### Sample size

Our sample size of 264 meets the recommended sample size of at least 200 for studies developing and testing instruments [22]. 

### Measures

#### DQI – Knowledge of lung cancer and LCS

Knowledge was measured at both T0 and T1 using 16 items (Table 1). Each knowledge item was recoded as ‘Correct’ (scored as 1) or ‘Incorrect’ (scored as 0, includes ‘don’t know’ responses). We calculated a total knowledge score using items that were retained after item retention and deletion. We developed a brief 5-item knowledge measure (LCS-5) focusing on knowledge of LCS guidelines (recommended screening frequency [KQ3]), LCS benefit (early detection [KQ9]), and LCS harms (false positives [KQ2], overdiagnosis [KQ8], and incidental findings [KQ14]). The sum of the correct responses to the knowledge items were standardized as a percentage and used in the analyses.

#### DQI – Goal concordance

Goal concordance was measured at T0 and T1 using 1 item. Following previous studies that assessed patient preferences directly [23, 24], we asked “Did you want to be screened for lung cancer?” (past tense used as all participants in this study underwent an LDCT). The response options were ‘I wanted to be screened’, ‘I wasn’t sure if I wanted to be screened’, and ‘I did not want to be screened’. Responses were recoded as ‘goal concordant’ (1; patient wanted to be screened and was screened) and ‘not goal concordant’ (0; patient was not sure or did not want to be screened but was screened).

#### DQI – SDM process (SDMP_4)

The quality of the SDM process was measured at T0 and T1 using SDMP_4 which had 4 items asking patients if their health care provider discussed the options (to screen or not be screened; Yes/No), pros of LCS (4-point response from ‘Not at all’ to ‘A lot’), cons of LCS (4-point response from ‘Not at all’ to ‘A lot’), and asked their preferences (Yes/No) [17]. The responses were recoded as binary responses and summed up for a total SDMP_4 score (range 0–4).

#### Decisional conflict (SURE)

SURE is a 4-item measure of decisional conflict measured at T0 and T1 [25]. The 4 items assess whether patients felt sure about the best choice for them (Sure of myself), they knew the benefits and risks of each option (Understand information), were clear about which benefits and risks matter most to them (Risk-benefit ratio), and have enough support and advice to make a choice (Encouragement) [25]. Response options were True (1) or False (0), and the items were summed up to create a SURE score (range 0–4), with higher scores indicating they were more sure about their choice, i.e. less decisional conflict. As each SURE item measures a different construct, some items were also analyzed individually in the validity hypotheses to assess face validity.

#### SDM quality (CollaboRATE)

CollaboRATE is a 3-item measure of SDM quality measured at T0 and T1 [26]. It measures the patient’s perception of the effort made by the clinician to help them understand their health issue, to listen to what matters to them with regards to their health issue, and to consider what matters most to them in the decision [26]. Each item was answered on a 10-point scale from ‘0’ (no effort was made) to ‘9’ (every effort was made). The mean of the three CollaboRATE items and the top scores (i.e., the percentage of participants who chose the highest score possible for all 3 CollaboRATE items) were calculated [26].

#### Decisional regret

At T0 and T1, we asked participants: “If you could make the decision about lung cancer screening again, what would you choose?”. The response options were recoded as ‘1’ (Definitely would make the same decision) or ‘0’ (Might make the same decision, might not make the decision, and definitely would not make the same decision).

#### Intentions to adhere to LCS guidelines

At T0 and T1, we asked participants: “Assuming your screening did not find anything abnormal, do you plan to be screened for lung cancer every year?”. The response options were recoded as ‘1’ (Definitely will be screened) or ‘0’ (Maybe will be screened, maybe will not be screened, and definitely will not be screened).

### Statistical analysis

Following the psychometric analysis of prior DQIs, [9, 11, 12] the criteria for evaluating patient-based outcome measures outlined by Fitzpatrick and colleagues was used to assess the psychometric properties of the DQI-LCS [27]. The statistical analyses mainly used T0 variables. T1 variables were used to establish test-retest reliability for the knowledge, screening preference, and decisional conflict variables. All available data was used in the analysis and participants with missing data were excluded.

#### Item retention and deletion

We examined the knowledge items for (1) index of discrimination [28, 29], (2) item difficulty, (3) item uncertainty, (4) corrected item-total correlation, and (5) inter-item correlation [28]. Table 2 below describes each of these psychometric tests and the benchmarks followed. We also examined floor or ceiling effects for the total knowledge score and total SDMP_4 score. Items that did not meet these benchmarks were flagged for further inspection but may be retained if they were a necessary fact for making an informed decision.

**Table 2:** Item retention and deletion – psychometric tests and benchmarks

Psychometric test	Description of test	Benchmarks
Index of discrimination	Assesses the validity of the items in discriminating between high and low performers in the sample, computed by taking the difference between the proportion of correct responses in the upper and lower 25th percentiles	> 40% – good item 30–39% – reasonably good item 20–29% – marginal item < 20% – poor item
Item difficulty	Percentage of participants who answered an item correctly	NA
Item uncertainty	Percentage of participants who answered “don’t know” on an item	NA
Corrected item-total correlation	Establishes consistency of the item with the total test score	< 0.2 – low consistency with other items
Inter-item correlation	Assess redundancy of items in a scale	> 0.8 – highly similar items

#### Feasibility

Feasibility was evaluated using the amount of missing data present in the T0 data. Items that had more than 5% missing data were considered for revision.

#### Test-retest reliability

To assess the test-retest reliability of the knowledge and SDMP_4 items, we calculated Cohen’s kappa for binary items and the intraclass correlation coefficient (ICC) for means, and aimed for Cohen’s kappa above 0.6 and ICC of at least 0.7 as an indicator of substantial reproducibility [30]. As the knowledge items do not measure a single latent construct, we did not calculate Cronbach’s alpha or evaluate internal consistency of the scale [31].

#### Validity of knowledge score

As there is no gold standard for validity of DQI instruments, we developed hypotheses to demonstrate the validity of the DQI-LCS instrument. To assess the construct validity of the knowledge component, we hypothesized that SURE scores would be positively associated with knowledge scores (H1a). In addition, we hypothesized that patients who felt they knew the benefits and risks of LCS (individual SURE item) will have greater knowledge than patients who did not or were not sure (H1b).

#### Validity of goal concordance

To assess the predictive validity of the goal concordance component, we hypothesized that patients who were goal concordant would have less decisional regret about getting screened (i.e. would make the same decision again) compared to those who were not goal concordant (H2). In addition, to assess the predictive validity of the goal concordance component, we also hypothesized that patients who were goal concordant would have higher intention to be screened again next year compared to those who were not goal concordant (H3).

#### Validity of SDMP_4

To assess concurrent validity, we hypothesized that the total SDMP_4 score should be strongly correlated (*r* > .7) with the CollaboRATE score (another measure of SDM quality) (H4). To assess the predictive validity of the SDMP_4 component, we also hypothesized that the SDMP_4 score would be positively associated with the SURE score (H5a). We also hypothesized that higher SDMP_4 scores should predict whether patients felt they had enough support and advice to make a choice (individual SURE item) (H5b).

## Results

### Sample characteristics

The final T0 sample (*N* = 264) was 48.0% female and had a mean age of 64.8. Overall, 92.8% (*n* = 245) of the patients in this study wanted to be screened and 6.8% (*n* = 18) were not sure if they wanted to be screened. Other sample characteristics have been reported previously [18, 20]. Of the 264 who completed the T0 survey, 230 completed the T1 survey (87.1% retention rate).

### Item retention and deletion

Table 3 shows the index of discrimination, item difficulty, item uncertainty, and corrected item-total correlation. Among the knowledge items, only one item, asking how much does screening lower the chances of dying from lung cancer (KQ13), had a poor index of discrimination (7.6%), indicating that this item poorly discriminated between high and low performers on knowledge. Only 2.7% of participants answered this item correctly. Hence, KQ13 was dropped. Three knowledge items, KQ10 (whether LCS is recommended for individuals with more than 15 years since quitting smoking), KQ11 (whether LCS is recommended for individuals with health problems that may shorten their life), and KQ16 (the amount of radiation exposure associated with the LDCT), had marginal indexes of discrimination (20–29%). A panel of experts in the study team, which included SDM experts and clinicians with LCS expertise, determined that these items had strong content relevance for evaluating whether patients are adequately informed about LCS. Hence, these three items were retained. With KQ13 dropped, we ended up with a 15-item knowledge of LCS scale (LCS-15) which was used in all analyses. Among the knowledge items, item difficulty ranged from 8.7% to 86.0% and item uncertainty ranged from 2.3 to 62.5%.

**Table 3:** Characteristics of knowledge items in the decision quality instrument for lung cancer screening (DQI-LCS)

	Individual knowledge Items	Index of discrimination^c^	Item difficulty^a^	Item uncertainty^b^	Corrected item-total correlation	Missing data	T0^d^ *N* (%) correct	T1^d^ *N* (%) correct	Cohen’s kappa^e^
KQ1	What is the best way to lower the chance of developing or dying from lung cancer?	47.0%	65.2%	2.3%	0.22	1.9%	172 (65.2)	149 (56.4)	0.43***
KQ2	How many people with an abnormal LCS result will have lung cancer?	36.4%	12.5%	62.5%	0.30	0.8%	33 (12.5)	26 (9.8)	0.44***
KQ3	How often do health professional groups recommend people get screened for lung cancer?	34.8%	48.6%	38.3%	0.11	0.4%	131 (49.6)	131 (49.6)	0.45***
KQ4	Where does lung cancer rank as a cause of cancer death in the US?	43.9%	29.5%	41.3%	0.19	0.4%	78 (29.5)	84 (31.8)	0.43***
KQ5	Do health professional groups recommend all current and former smokers be screened for lung cancer?	37.9%	19.7%	22.7%	0.25	0.4%	52 (19.7)	32 (12.1)	0.44***
KQ6	Can lung cancer screening suggest that you have lung cancer when you do not?	71.2%	44.3%	43.2%	0.45	0%	117 (44.3)	118 (44.7)	0.51***
KQ7	Can lung cancer screening miss a possible lung cancer?	80.3%	58.3%	37.5%	0.50	0.4%	154 (58.3)	139 (52.7)	0.46***
KQ8	Will all tumors found by lung cancer screening grow to be life threatening?	74.2%	64.0%	31.1%	0.47	0%	169 (64.0)	149 (56.4)	0.46***
KQ9	Without screening, is lung cancer often found at a later stage when cure is less likely?	34.8%	86.0%	12.5%	0.31	0%	227 (86.0)	201 (76.1)	0.35***
KQ10	Is screening recommended for someone who quit smoking more than 15 years ago?	28.8%	8.7%	25.4%	0.35	0%	23 (8.7)	23 (8.7)	0.39***
KQ11	Is screening recommended for someone who has other health problems that may shorten their life?	24.2%	10.2%	39%	0.22	0%	27 (10.2)	23 (8.7)	0.35***
KQ12	Is screening recommended for someone who is not able or willing to be treated for lung cancer?	48.5%	28.8%	42.8%	0.24	0%	76 (28.8)	68 (25.8)	0.32***
KQ13 *[dropped]*	How much does screening for lung cancer lower the chances of dying from lung cancer? *[dropped]*	7.6%	2.7%	54.9%	NA	0%	7 (2.7)	9 (3.4)	0.38***
KQ14	Can lung cancer screening find other health conditions?	59.1%	79.5%	18.9%	0.46	0%	210 (79.5)	189 (71.6)	0.49***
KQ15	What is the leading cause of lung cancer in the United States?	40.9%	84.1%	9.1%	0.26	0.4%	222 (84.1)	204 (77.3)	0.45***
KQ16	How much radiation is a person exposed to through lung cancer screening?	28.8%	9.5%	45.5%	0.29	0.4%	25 (9.5)	26 (9.8)	0.56***

Note: *P < .05, **P < .01, ***P < .001

^a^Item difficulty is the percentage of people who answer an item correctly

^b^Item uncertainty is the percentage of people who answered “don’t know” to the question

^c^Index of discrimination is calculated as the difference between the proportion of correct responses of participants in the upper and lower 25th percentiles

^d^The T0 and T1 means were calculated using recoded binary responses

^e^Cohen’s kappa was used to calculate test-retest reliability. We used the recoded correct, incorrect, and unsure responses

In the T0 LCS-15 scale, two items (KQ3 and KQ4) had corrected item-total correlations under 0.2, indicating that they were not highly consistent with the rest of knowledge items in the scale. In the T0 LCS-5, two items (KQ2 and KQ3) had corrected item-total correlations under 0.2. There were no inter-item correlations above 0.8 for both LCS-15 (Table 4) and LCS-5 (Table 5), indicating low redundancy within the scales. In summary, in both the LCS-15 and LCS-5 scales, we found that there was low overlap between items but a few items were not highly consistent with the others, which was expected since the knowledge items do not measure a single underlying construct.

**Table 4:** Inter-item correlation for T0 LCS-15 (full lung cancer screening knowledge scale)

	KQ1	KQ2	KQ3	KQ4	KQ5	KQ6	KQ7	KQ8	KQ9	KQ10	KQ11	KQ12	KQ14	KQ15	KQ16
KQ1	1.00														
KQ2	0.07	1.00													
KQ3	0.06	0.02	1.00												
KQ4	0.04	0.07	0.16	1.00											
KQ5	0.01	0.15	0.01	0.08	1.00										
KQ6	0.11	0.19	0.11	0.05	0.10	1.00									
KQ7	0.18	0.20	0.06	0.09	0.15	0.54	1.00								
KQ8	0.18	0.23	0.02	0.07	0.17	0.37	0.41	1.00							
KQ9	0.08	0.05	0.04	0.16	0.11	0.11	0.20	0.18	1.00						
KQ10	0.14	0.21	0.15	0.07	0.23	0.24	0.18	0.12	0.05	1.00					
KQ11	− 0.02	0.14	0.06	0.03	0.16	0.13	0.08	0.18	0.14	0.20	1.00				
KQ12	0.11	0.12	− 0.06	0.003	0.17	0.12	0.15	0.11	0.18	0.25	0.17	1.00			
KQ14	0.14	0.16	0.06	0.15	0.17	0.30	0.45	0.36	0.26	0.12	0.04	0.19	1.00		
KQ15	0.20	0.13	0.06	0.18	0.04	0.12	0.13	0.21	0.24	0.06	0.01	− 0.02	0.17	1.00	
KQ16	0.13	0.19	− 0.02	0.08	0.14	0.24	0.12	0.22	0.13	0.17	0.14	0.14	0.13	0.07	1.00

Note. Q13 was dropped as it had a poor index of discrimination, indicating that it discriminated poorly between respondents who scored high and low on knowledge and may too difficult for most respondents

**Table 5:** Inter-item correlation for T0 LCS-5 (brief lung cancer screening knowledge scale)

	KQ2	KQ3	KQ8	KQ9	KQ14
KQ2	1.00				
KQ3	0.01	1.00			
KQ8	0.24	0.03	1.00		
KQ9	0.06	0.03	0.18	1.00	
KQ14	0.13	0.09	0.36	0.23	1.00

### DQI – Knowledge score

The mean knowledge score using LCS-15 at T0 was 43.3% (SD = 18.1, range 0-93.3%) and 45.3% at T1 (SD = 16.7, range 6.7–100%). Using the brief LCS knowledge measure (LCS-5), the mean knowledge score was 58.3% (SD = 23.1) at T0 and 60.5% (SD = 22.6) at T1. The range of LCS-5 was 0% to 100% at both T0 and T1. The percentage scores of LCS-15 and LCS-5 were highly correlated at T0 (*r* = .82, *P* < .001) and T1 (*r* = .73, *P* < .001). There was no evidence of a floor or ceiling effect at either survey time-point with visual inspection of histograms.

### DQI – Goal concordance

Most participants (92.8%) were goal concordant, while 6.8% were not goal concordant (reported being unsure about undergoing LCS but had LCS).

### DQI – SDM process score (SDMP_4)

For SDMP_4 (theoretical range = 0–4), the mean at T0 was 2.2 (SD = 1.3) and the mean at T1 was 2.5 (SD = 1.4). No floor or ceiling effects were found.

### Feasibility

None of the knowledge, goal concordance, or SDMP_4 items had more than 5% missing data (Table 3).

### Test-retest reliability

For the individual knowledge items, Cohen’s kappa ranged from 0.3 to 0.6 (all *P* < .001). The ICC was 0.7 (*P* < .001) for the total LCS-15 score, and 0.7 (*P* < .001) for the total LCS-5 score. Screening preference, which had only 1 item, had a Cohen’s kappa of 0.5 (*P* < .001). For the individual SDMP_4 items, Cohen’s kappa ranged from 0.3 to 0.5 (all *P* < .001). The ICC for the total SDMP_4 score was 0.6 (*P* < .001).

### Validity

#### Knowledge

The mean of the full SURE scale was 3.09 (SD = 1.47, range 1–4). The overall regression model of SURE scores predicting LCS-15 knowledge was statistically significant (*R*^*2*^ = 0.03, *F*[1,258] = 8.29, *P* < .01). An increase in SURE scores was positively associated with an increase in LCS-15 knowledge (*b* = 0.18, *P* < .01). The regression model using LCS-5 knowledge showed the same results (*R*^*2*^ = 0.07, *F*[1,258] = 18.11, *P* < .001; *b* = 0.26, *P* < .001). As predicted, feeling more sure about their choice (i.e., having less decisional conflict) was associated with better LCS knowledge; H1a was supported.

Of participants, 70.8% reported that they were clear about the benefits and risks of LCS that matter most to them (individual SURE item), and 27.6% were not sure or were not clear. Participants who reported that they knew the benefits and risks of LCS had a significantly higher mean LCS-15 knowledge score (M = 45.0%, SD = 16.8) compared to participants who did not or were unsure (M = 39.1%, SD = 20.8) (Mean difference = 5.9, *P* < .05, 95% CI 1.0, 10.9, *d* = 0.31). A similar finding was found using LCS-5. Participants who reported that they knew the benefits and risks of LCS had a significantly higher mean LCS-5 knowledge score (M = 61.5%, SD = 20.7) compared to participants who did not or were unsure (M = 50.1%, SD = 27.1) (Mean difference = 11.3, *P* < .001, 95% CI 5.1, 17.5, *d* = 0.47). Hence, awareness of the benefits and risks of LCS was associated with better LCS knowledge. H1b was supported and there was a small to medium effects size [32].

#### Goal concordance

Among participants, 86.4% (*n* = 228) reported that they would definitely make the same decision again (i.e., low decision regret), and 12.2% might or definitely would not make the same decision again. When the participant was goal concordant, we found that there was a greater probability of the participant reporting that they would make the same decision again (OR = 8.1, 95% CI 2.9, 23.0). In other words, when participants received screening that they wanted, there was less decision regret. H2 was supported.

We found that 66.7% (*n* = 176) of participants reported that they would definitely be screened every year (i.e., intentions to adhere to annual LCS) and 31.8% said that they either may be screened, may not be screened, or definitely would not be screened every year. We found that participants who were goal concordant had a greater probability of reporting that they intended to be screened again the next year (OR = 7.3, 95% CI 2.3, 23.3). In other words, when participants received screening that they wanted, they had greater intentions to adhere to annual LCS. H3 was supported.

#### SDM process score (SDMP_4)

The mean CollaboRATE score was 78.8 (SD = 25.7) at T0 and 81.9 (SD = 21.6) at T1. CollaboRATE top scores were 35.6% and 29.9% of participants at T0 and T1 respectively. At T0 and T1, the correlation between the total SDMP_4 score and CollaboRATE did not exceed the expected correlation of 0.7 (both *r* = .5, *P* < .001). The correlations at T0 and T1 were significantly different from the expected value (*z*=-5.159, *P* < .001). H4 was not supported.

The regression model with SDMP_4 predicting SURE scores was statistically significant (*R*^*2*^ = 0.21, *F*[1,256] = 66.97, *P* < .001). We found that an increase in SDMP_4 scores was positively associated with an increase in SURE scores (*b* = 0.46, *P* < .001). H5a was supported. When SDMP_4 scores increased, there was a greater probability that the participant reported feeling like they had enough support and advice to make a choice (OR = 2.6, 95% CI 1.9–3.4). H5b was also supported. In summary, when participants reported a higher quality SDM process, this was associated with less decisional conflict and greater likelihood that they felt supported in making a decision.

## Discussion

Overall, the DQI-LCS was feasible to complete but test-retest reliability was moderate for most components. Majority of our validity hypotheses were supported, indicating acceptable construct and predictive validity of the DQI-LCS.

The first DQI-LCS component, knowledge of LCS (LCS-15), is adapted from a validated measure of LCS knowledge [16, 33]. There have been other instruments measuring knowledge of the risks and benefits of LCS, but their psychometric properties have not been reported [34–36]. To enhance the clinical utility of the DQI-LCS, we also developed a brief knowledge measure (LCS-5) that was highly correlated with the LCS-15, suggesting that the LCS-5 can be used as an effective measure of LCS knowledge in settings where the full scale cannot be feasibly implemented. As expected, participants who had higher SURE scores or who reported being aware of the benefits and harms of LCS were also more knowledgeable about LCS, demonstrating acceptable construct validity of both the LCS-15 and LCS-5.

Overall knowledge about LCS were low in our sample. In particular, most participants did not know how many people with an abnormal LCS result will have lung cancer, if screening is recommended for those who quit smoking and for patients who have other health conditions, and the radiation exposure associated with LCS. Considering that all participants had already received LCS, our findings suggest that patients may not be fully informed about the harms and benefits of LCS prior to making a decision. This is aligned with extant research showing that there are known gaps in patients’ knowledge about LCS, due to barriers such as clinicians’ lack of time during visits to conduct SDM, clinicians’ lack of resources (e.g. tools, training) to support conducting high-quality SDM, and a tendency to emphasize screening benefits and not harms [37–39]. Mean knowledge scores were higher when using the LCS-5, which only included items considered essential for making an LCS decision, but were still approximately 60% at both T0 and T1. This indicates that some knowledge items may have been challenging for patients to answer because they are not commonly covered during LCS conversations; however, it is still concerning that participants did not appear to be well-informed about basic information about the benefits and harms of LCS, as required by CMS [5]. It is also possible that patients may not have retained the information about LCS because they had already made a decision and committed to a long-term plan. For example, a study of patients who had recently (up to 1 month prior) had an SDM discussion using a decision aid with a dedicated screening coordinator found that only 44% answered the basic knowledge question correctly [40]. This suggests that even when patients were well-informed at the time of decision-making, they did not retain information over time [40]. To obtain a more accurate estimate of LCS knowledge, future research should consider administering the DQI-LCS at the time of the screening decision.

Few patients in our sample received screening that they did not explicitly want. This finding is also in line with extant research; a few studies have reported that patients in the US find LCS acceptable and were likely to talk to their doctor about LCS [41, 42]. We also found that when a participant was goal concordant, they were less likely to have decisional regret and more likely to intend to adhere to annual LCS guidelines. Other DQIs have similarly found that patients who were concordant had less decisional regret [12, 43]. However, few studies have examined the relationship between goal concordance and intentions to adhere to screening. Given that adherence to LCS in the US is estimated to be between 22.3% to 55%, [44, 45] this finding highlights the potential importance of ensuring goal concordance for patients to enhance adherence to annual LCS. That said, as we had a very small number of participants who were *not* goal concordant, we are cautious in interpreting the odds ratios from our analysis. Future studies should include patients who were eligible for LCS but decided not to be screened to further test the predictive validity of the goal concordance component.

We utilized the SDMP_4 as our measure of the quality of the SDM process in our DQI-LCS [17]. The SDMP_4 has been validated across different clinical decisions [10, 12, 23, 46]. To our knowledge, SDMP_4 has only been used in one published LCS study, [18] making this the first psychometric evaluation of SDMP_4 for LCS. In terms of construct validity, the correlation of SDMP_4 with the CollaboRATE measure (*r* = .5) did not meet the threshold we hypothesized, but was similar to correlations found in other studies on hip/knee and back treatment decisions (*r* = .4) [47]. This may be because the two measure different constructs; SDMP_4 is a measure of the presence or absence of four key SDM elements during the clinical encounter, [46] while CollaboRATE is a measure of the patient’s perception of the amount of effort made by the clinician to engage them in SDM [26]. Despite not meeting the correlation threshold, SDMP_4 and CollaboRATE were still strongly correlated, suggesting that that they were conceptually different but related measures of SDM quality. Additionally, it is possible that the limited range of responses typically seen with CollaboRATE scores may have reduced the strength of the observed correlation. Finally, in previous studies, higher SDMP_4 scores have been significantly associated with higher SURE scores [10, 17]. We similarly found that higher SDM process scores predicted higher SURE scores and led to participants feeling that they had enough support and advice to make a choice, further highlighting the importance and positive impact of a high quality SDM process for patients.

The overall knowledge score (both LCS-15 and LCS-5) had acceptable test-retest reliability. The individual knowledge items had moderate test-retest reliability, and screening preference, the individual SDMP_4 items, and the full SDMP_4 scale had fair to moderate test-retest reliability. These findings may be explained by recall bias in our study, as we conducted retrospective surveys where participants were asked to recall a decision made up to 12 months ago, with one month in between the baseline and follow-up surveys. Prior studies which found acceptable test-retest reliability of the SDMP_4 had also conducted retrospective surveys for decisions made in the past 1–2 years; [10, 23] however, these had follow-up surveys conducted 1–2 weeks after the initial survey, [10] which may have aided recall. Hence, the combination of a long recall period and long follow-up of 1 month may have contributed to the lower than expected test-retest reliability scores in our study. Future research in this area can potentially improve test-retest reliability by conducting additional rounds of cognitive testing of the instrument with a diverse set of participants, [48] modifying responses options of highly difficult items to Yes/No or True/False formats rather than a multiple choice format, and administering the DQI-LCS instrument closer to the screening decision to minimize recall bias and allow for more accurate assessment of the instrument’s performance.

CMS requires that a counseling and SDM visit take place prior to screening, but there is evidence that many of these conversations do not occur or do not address essential elements of SDM [20, 49, 50]. There are several ways that the DQI-LCS can be used in clinical settings. First, the DQI-LCS can be used as a way to audit overall LCS decision quality in primary care settings. For instance, the DQI-LCS could be administered (e.g., paper questionnaire completed in the waiting room or online questionnaire sent via a electronic health record message) after a visit where the SDM conversation about LCS occurred. Findings from the questionnaire could be used to assess knowledge levels after the SDM discussion, quality of the SDM process, and establish goal concordance with screening behavior. This could help identify common gaps in decision quality (e.g. essential LCS information not provided), serve as feedback for clinicians or other clinical staff conducting SDM, and inform quality improvement projects to support primary care clinicians in bridging these gaps. Pre- and post-visit use of the DQI-LCS could also be used to evaluate efficacy of quality improvement projects or decision support interventions (e.g. use of a decision aid or decision coaching with a patient navigator) [51–54]. Second, the knowledge and goal concordance components of the DQI-LCS could be administered to patients before the clinic visit to identify gaps in knowledge and understand screening preferences. This information could then be used to streamline discussions with the clinician to allow for more efficient use of the visit time (e.g. focusing the discussion on knowledge gaps or misconceptions about LCS, and reasons for their screening preferences). Third, with routine incorporation of the DQI-LCS in the clinical workflow, improvements over time could be monitored and used to establish feasible benchmarks of LCS decision quality in primary care settings.

Our study has several limitations. First, patient acceptability of the DQI is typically measured using survey response rates [12]. However, as survey packets were distributed to potential participants by our collaborating clinical sites and then mailed back to the study team by participants, we were not able to calculate the response rate. Second, we only included patients who had undergone LCS, limiting the interpretation of our goal concordance analysis as we had a very small number of individuals who were not goal concordant. A larger sample size of individuals who are not goal concordant (i.e., inclusion of patients who wanted to be screened but were not screened) would allow us to obtain more accurate estimates of the impact of goal concordance on decision regret and intentions to engage in future LCS. Given that patients who wanted to be screened but were not screened likely face systemic barriers to screening (e.g., lack of insurance, transportation, time, and cost barriers), [55] it remains unclear how this impacts decision regret and intentions to screen in the future. Future research should also explore if patients who were unsure about LCS and declined LCS (goal concordant) experienced decision regret and intended to screen in the future. Third, previous psychometric evaluations of DQIs have also assessed validity of the instrument by examining whether participants’ top goals and concerns were predictive of their decision to undergo the treatment or not, which we were not able to do in this paper as all participants underwent LCS. Finally, the DQI-LCS is currently only available in English. Previous studies have demonstrated that individuals who have limited English proficiency may experience lower perceived patient-centered care and communication quality, and that language-concordant care is associated with better health outcomes [56, 57]. This underscores the importance of evaluating LCS decision quality among linguistically diverse populations using standardized instruments. Future studies should culturally and linguistically adapt and test the DQI-LCS in other languages for individuals with limited English proficiency.

## Conclusion

LCS has both potential benefits and harms for patients, and it is crucial that patients are adequately informed and that their preferences are considered prior to being screened. To our knowledge, this is the first decision quality instrument developed for LCS decision and the only DQI-LCS instrument that has undergone rigorous psychometric testing. The DQI-LCS was shown to be feasible for participants to complete, had moderate test-retest reliability, and had acceptable construct and predictive validity. Hence, it can serve as a useful tool to assess LCS decision quality (i.e., if patient’s LCS knowledge prior to screening, whether they are undergoing a high quality SDM process when deciding whether to screen for lung cancer, and whether their choice is concordant with their goals). In addition, a brief DQI-LCS with a 5-item measure of LCS knowledge has been developed and psychometrically test to enhance its clinical utility. Future research should continue to test and validate the psychometric properties of the DQI-LCS with different populations, including people who went through the SDM process but decided against LCS.

## Data Availability

The data that support the findings of this study are available from the corresponding author upon reasonable request.

## Abbreviations

LCS: Lung cancer screening
USPSTF: US Preventive Services Task Force
CMS: Centers for Medicare & Medicaid Services
SDM: Shared decision-making
IPDAS: International Patient Decision Aids Standards
DQI: Decision quality instrument
DQI-LCS: Decision quality instrument for lung cancer screening
SDMP_4: Shared Decision Making Process Measure
ICC: Intraclass correlation coefficient
